# Editorial: Natural products as drivers in drug development for neurodegenerative disorders

**DOI:** 10.3389/fphar.2022.932179

**Published:** 2022-08-04

**Authors:** Joana Silva, Rebeca Alvariño, Márcia I. Goettert, Hector J. Caruncho, Celso Alves

**Affiliations:** ^1^ MARE—Marine and Environmental Sciences Centre, Polytechnic of Leiria, Peniche, Portugal; ^2^ Department of Pharmacology, Faculty of Veterinary, University of Santiago de Compostela, Lugo, Spain; ^3^ Department of Pharmaceutical and Medicinal Chemistry, Institute of Pharmacy, Eberhard Karls Universität Tübingen, Tübingen, Germany; ^4^ Cell Culture Laboratory, Postgraduate Programme in Biotechnology, University of Vale do Taquari Univates, Lajeado, Brazil; ^5^ Division of Medical Sciences, University of Victoria, Victoria, BC, Canada

**Keywords:** natural products (NP), neuroinflammation, oxidative stress, apoptosis, mechanisms of action, neuropharmacology, drug discovery, ethnopharmacology

The prevalence of neurological disorders (NDs) is a large and increasing health burden worldwide and is one of the emerging reasons for morbidity, mortality, and cognitive impairment in aging. Impact of NDs is expected to significantly increase in the next decades due to the progressive aging of the world society (Feigin and Vos, 2019). In the specific case of neurodegenerative process, it leads to malfunctions and cellular death, which seems to be triggered by a set of complex biological mechanisms, such as protein aberrant aggregation, mitochondrial dysfunction, oxidative stress, defective protein quality control, and degradation pathways, stress granules, and maladaptive immune response (Moujalled et al., 2021). Despite the advances achieved, the effectiveness of current drugs to control, delay, or block the NDs progression is still limited (Durães et al., 2018). Thus, the discovery and development of new therapeutic agents that can improve the currently therapeutic regimes are of utmost importance. Accordingly, a large focus has been placed on the potential of natural products (NPs) as new natural neuroprotective agents, essentially due to their scaffold diversity, structural complexity, and ability to activate several intracellular signaling pathways through distinct mechanisms of action while presenting fewer side-effects (Alghamdi et al., 2022).

Over the last decades, nature has revealed to be a prolific source of unparalleled structurally active metabolites, with a broad spectrum of biological activities, including antibacterial, antifungal, anticancer, antifouling, antioxidant, anti-inflammatory, and neuroprotective properties (Grkovic et al., 2014; Newman and Cragg, 2020; Atanasov et al., 2021; Singla et al., 2021). To date, quite a wide range of NPs classes have been isolated from terrestrial and marine organisms, including alcohols, alkaloids, amino acid derivatives, aromatic compounds, fatty acids, lactones, peptides, polyacetylenes, polyketides, quinones, quinolones, sphingolipids, sterols, terpenes, and terpenoids, among others (Newman and Cragg, 2020). Since those metabolites are produced as a response to physical and ecological pressures to ensure organisms survival and have presumably evolved over billions of years in close association with biological systems, they present high specificity and great affinity to interact with biological target structures, making them excellent candidates to inspire the development of new medicines, with minimal collateral effects and significant health benefits (Hong, 2011; Li et al., 2019).

The Research Topic here presented was focused on the potential of natural products (NPs) as scaffolds to inspire the development of new drugs for neurodegenerative disorders treatments, emphasizing the diversity of molecular targets and mechanistic effects. This topic gathered a collection of three critical reviews and four original research articles, with over 10,800 views and more than 2,200 article downloads, highlighting the capacity of NPs to target key players of signaling pathways related with NDs development, namely Alzheimer’s disease (AD), depression, and infectious diseases.

Fakhri et al. provide a review regarding the neuronal symptoms of emerging infectious diseases (EID) and underlying pathophysiological mechanisms. The capacity of NPs acting as multi-target agents on dysregulated pathways towards neuroprotection related with EID was investigated applying a mechanistic-based strategy.

Nowak et al. reported a detail review regarding the neuroprotective properties of *Ginkgo biloba L* [Ginkgoaceae] and its most used standardized extract (EGb 761) on the intracellular signaling pathways related with AD pathogenesis hallmarks.

On the other hand, Piccialli et al. focused their review on the therapeutic potential of polyphenols and monoterpenes, two of the major chemical classes of natural products, in AD, reporting the intracellular signaling pathways activated by them.

Glycogen synthase kinase 3β (GSK3β) pathway have been associated to the pathophysiology of AD. Its permanent abnormal activation compromises the synaptic signals critical for learning and memory processes in AD, leading to memory impairment, increased production of Aβ, and inflammatory responses (Lauretti et al., 2020). Rodríguez-Urgellés et al. described the *in vivo* neuroprotective potential of meridianins, marine indole alkaloids, as GSK3β inhibitors. The treatments performed in 5xFAD mice exhibited a marked improvement of recognition memory and cognitive abilities avoiding the synaptic loss and reduced the neuroinflammatory processes.

The higher beneficial effects of trans ε-viniferin, a natural polyphenol biosynthetized by *Vitis vinifera L* [Vitaceae], have been suggested comparing with the standard polyphenol resveratrol, due to its chemical structure and reduced catabolism (Boocock et al., 2007). Freyssin et al. reported the higher neuroprotective effects of viniferin compared to resveratrol an *in vivo* model of AD after a prolonged exposure, reducing amyloid load and deposits, partly preventing the cognitive decline and being more effective against glial reactivity.

Pan et al. studied the *in vivo* antidepressant effects of total triterpenes attained from a sclerotium of the polyporaceae fungus *Wolfiporia cocos* (TTWC) commonly used in traditional Chinese medicine to treat depression. TTWC treatment induced an antidepressant-like effect regulating the levels of neurotransmitters, hypothalamic-pituitary-adrenal (HPA) axis, and NLRP3 signaling pathway.

Yang et al. investigated the ability of Chinese medicinal plants (CMP) components to target serotonin receptor 5-HT1B, which is widely expressed in several of cognitive and psychiatric disorders, such as depression and AD (Gadgaard and Jensen, 2020). Emodin-8-O-β-d-glucopyranoside (EG) active ingredient displayed to be a selective agonist of 5-HT1B activating 5-HT1B-induced signaling pathway and modulating Aβ-related inflammatory process regulation and neural death resistance.

Altogether, the seven publications gathered in this topic provide a relevant overview of natural products as potential therapeutic neuroprotective agents, with special focus in AD, depression, and neuronal symptoms related with emerging infectious diseases.
